# High prevalence of burnout syndrome in Czech general practitioners: A cross-sectional survey

**DOI:** 10.1016/j.pmedr.2023.102502

**Published:** 2023-11-10

**Authors:** Ladislav Štěpánek, Mihir Sanjay Patel, Dagmar Horáková, Lubica Juríčková, Svatopluk Býma

**Affiliations:** a Department of Public Health, Faculty of Medicine and Dentistry, Palacký University Olomouc, Olomouc, Czech Republic; b Institute of Preventive Medicine, Faculty of Medicine in Hradec Králové, Charles University, Hradec Králové, Czech Republic

**Keywords:** Burnout, General practitioner, Prevalence, Emotional exhaustion, Depersonalization, Personal accomplishment, Job demands-resources model

## Abstract

**Objective:**

A wide range in prevalence rates of burnout among general practitioners (GPs) has been reported in various regions, with an increasing trend. This nationwide cross-sectional study aimed to estimate the prevalence and associated determinants of burnout in Czech GPs.

**Methods:**

1000 randomly selected physicians from the Czech Society of General Practitioners (through a pseudorandom number generator) were emailed an online survey based on the Maslach Burnout Inventory – Human Services Survey. Data collection was performed between January and February 2023.

**Results:**

331 questionnaires were obtained (227 females and 104 males, mean age – 49.9 years, the mean number of registered patients – 1951). 21.8 % of GPs scored a high level of burnout in all three of its dimensions and 23.9 % in no dimension at all. The most prevalent dimension was reduced personal accomplishment (PA, 56.2 %) followed by emotional exhaustion (EE, 50.2 %) and depersonalization (DP, 40.5 %). Reaching burnout in all three dimensions was significantly more frequent in males and in GPs registering a number of patients above the median. Increasing age and years of practice were protective factors for DP but risk factors for reduced PA. Employed GPs had lower EE scores than GP practice owners. The respondents' basic characteristics reflected their presence among Czech GPs, which testifies against selection bias.

**Conclusions:**

The high rate of burnout (∼22 %) should be addressed by promoting personal resources along with the perception of the importance of GPs in society. A sufficiently dense network of GPs should allow them to register a lower number of patients.

## Highlights

• Severe burnout concerns 21.8% of Czech general practitioners.
• The most prevalent dimension of burnout is a lack of personal accomplishment.
• 23.9% of Czech general practitioners do not present with burnout in any dimension.
• Burnout prevalence increases with the number of registered patients.
• Awareness of the importance of general practitioners should be raised in society.

## 1 Introduction

Burnout is a psychological syndrome caused by the long-term accumulation of occupational stress. The three key dimensions of this syndrome are emotional exhaustion (EE - depletion of mental resources caused by excessive frustration or fear of work), depersonalization (DP - negativity and apathy towards work, difficulty concentrating on work or lack of initiative or enthusiasm), and decreased personal accomplishment (PA - a diminished sense of occupational accomplishment, feelings of incompetence or uselessness to society) (Maslach and Leiter, 2016, Shen et al., 2022). Physician burnout is associated with negative consequences on patient care (lower quality of care, medical errors and lower patient satisfaction), physician workforce, healthcare system costs, and physicians’ safety (West et al., 2018). A wide range of prevalence rates of burnout among general practitioners (GPs) has been reported in various regions indicating a progressive increase in the prevalence of burnout (Brown et al., 2019, Karuna et al., 2022). This study aimed to estimate the prevalence and associated determinants of burnout with its dimensions in Czech GPs.

## 2 Methods

A priori power analysis was performed applying inference test design and asymptotic test properties using two assumptions; (1) a constant population effect size (Maslach et al., 1997, Meier and Kim, 2022), and (2) significance level alpha = 0.05 and statistical power beta = 0.05, respectively. A minimum sample size of 108 survey responses was determined for the study. Previous surveys of a similar nature (Štěpánek et al., 2023) yielded a ∼ 12 % response rate which helped establish at least 900 potential participants as a sufficient sample size. The Czech Society of General Practitioners, which unites GPs in the Czech Republic (n = 4,800), randomly selected 1,000 members, to whom an email request to participate in the survey was sent on January 12, 2023. Subsetting using a pseudorandom number generator was applied in the random selection. Data collection ended on February 28, 2023. The questionnaire consisted of two parts: Initially, 5 mostly closed-ended questions on the respondent’s age, sex, duration of practice as a GP, number of registered patients and ownership/employment of a GP practice. The second part involved the Maslach Burnout Inventory – Human Services Survey (MBI-HSS), including 22 items grouped into 3 subscales according to the 3 dimensions of burnout (9 items for EE, 5 items for DP and 8 items for PA). Each item was scored on a Likert scale, from never (0) to every day (6).

Statistical analysis was carried out with the SPSS software (version 22.0). Besides descriptive statistics, Spearman’s correlation coefficients were determined between numerical variables and burnout scores. Cronbach’s alpha was calculated to assess the internal consistency of data. A p-value lower than 0.05 indicated statistical significance. The MBI scores for each dimension were assessed separately and recommended thresholds for a low, moderate, or high level of burnout were applied for every subscale. High EE, high DP, and low PA were considered unfavourable score ranges and indicated burnout in each dimension (Karuna et al., 2022, Maslach et al., 1997).

## 3 Results

331 completed questionnaires were obtained from 227 females and 104 males with a mean age of 49.9 years (median 49). Respondents worked as a GP for an average of 18.6 years (median 15), caring for 1950.6 patients (median 1900). 79.8 % of participating GPs owned their practice, while 20.2 % were employed.

21.8 % of respondents achieved burnout in all three dimensions. Conversely, 23.9 % of GPs did not present with burnout in any dimension, and 27.2 % identically in one and two subscales. The most prevalent dimension reaching burnout was PA, found in 56.2 % of respondents. This was followed by EE in 50.2 % and DP in 40.5 % of respondents (Table 1). EE and DP scored low levels in ∼ 34 % of the respondents, while the high level of PA, indicating a lower extent of burnout, affected only 18 % of GPs.

**Table 1:** Prevalence of each burnout dimension in Czech general practitioners (n = 331).

	Emotional exhaustion	Depersonalization	Personal accomplishment
**Burnout levels**			
Low level (N (%))	115 (34.7)	113 (34.1)	186 (56.2)
Moderate level (N (%))	50 (15.1)	84 (25.4)	84 (25.4)
High level (N (%))	166 (50.2)	134 (40.5)	61 (18.4)
**Burnout scores**			
Mean (95 % confidence interval)	26.4 (24.9 – 27.8)	8.9 (8.1 – 9.6)	31.5 (30.6 – 32.5)
Median	41	14	43

**Fig. 1:**
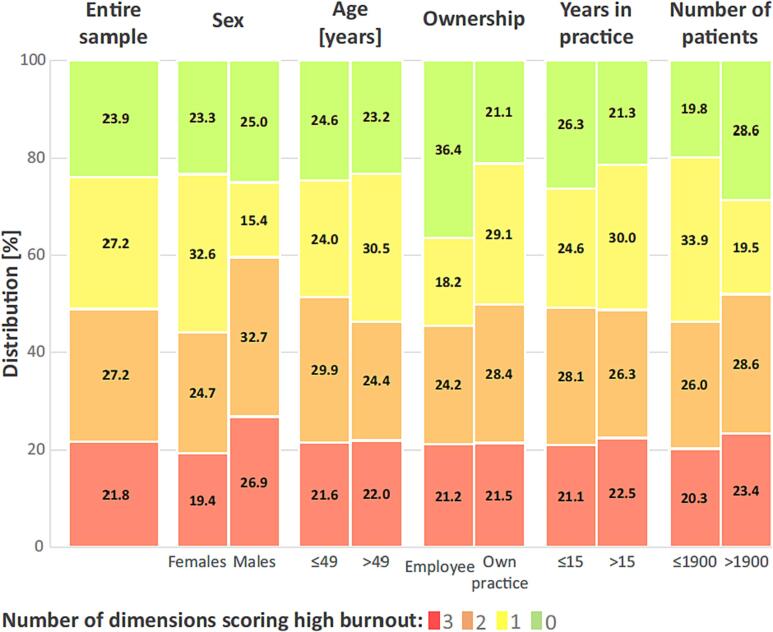
Representation of Czech general practitioners reaching a high score of burnout in a particular number of dimensions (from 0 to 3) in the entire sample and subgroups.

Males and GPs registering more patients than the sample median scored significantly more often burnout in all three dimensions (Fig. 1). Regarding scores in individual subscales, males achieved a statistically significantly higher mean value in DP than females (10.63 ± 7.3 vs. 8.04 ± 6.28, p = 0.001). Respondents who owned their practice had higher EE scores when compared with employed respondents (27.16 ± 13.83 vs. 23.3 ± 11.33, p = 0.037). Correlation analysis revealed that with increasing age and number of years in practice, the score of DP (r = -0.12, p < 0.001 and r = -0.18, p = 0.009, respectively) and PA (r = -0.18, p < 0.001 and r = -0.11, p = 0.003, respectively) decreased. Increased age and years in practice presented as protective factors for DP but possible risk factors for reduced PA. The Cronbach’s alphas for the MBI-HSS, EE, DP, and PA were 0.85, 0.94, 0.8 and 0.84, respectively.

## 4 Discussion

Globally, the reported prevalence of burnout in GPs is highly variable, with rates ranging between 2.8 % and 85.7 % (McCammon et al., 2023). MBI–HSS is the most frequently applied instrument to measure burnout prevalence, although methods of data interpretation are inconsistent (Karuna et al., 2022, McCammon et al., 2023). A MBI-pattern refers to various permutations of single and combined MBI dimensions of the three score ranges and relates to how MBI results are presented. The available literature consists of a variety of different MBI-patterns. The most unfavourable MBI-pattern (high EE, high DP and low PA) is typically called severe burnout or a high overall degree of burnout (Karuna et al., 2022, McCammon et al., 2023). 21.8 % of respondents in our study presented with this classification of severe burnout. Recent studies in other countries detected lower levels of severe burnout in GPs – 4.8 % in France (n = 1926) and 7.5 % in Germany (n = 214) (Dreher et al., 2019, Dutheil et al., 2021). A meta-analysis summarizing the available global literature showed that 37 %, 28 %, and 26 % of GPs suffered from high EE, DP, and low PA, respectively (Shen et al., 2022). Czech GPs seem to be significantly more burnt out when compared to foreign doctors, especially in PA. However, the previously mentioned studies reflect the situation before the COVID-19 pandemic. An increase in job demands and mental health disorders emerged with the COVID-19 pandemic among ambulatory care workers (Biber et al., 2022, Mojtahedzadeh et al., 2021).

Males showed severe burnout more often than females, which complies with the cited French study (Dutheil et al., 2021). On the contrary, in the study from Germany, females tended to present with higher levels of burnout (Dreher et al., 2019). Increasing age was a protective factor for DP and a risk factor for low PA in the present study. However, age was not a risk factor for overall severe burnout (Fig. 1) as indicated also in the French study (Dutheil et al., 2021).

Occupation-specific determinants play a significant role in acquiring burnout when compared to generic determinants (Verhoef and Blomme, 2022). Our study indicated that GPs registering a greater number of patients were more inclined to experience severe burnout. Some European regions, including the Czech Republic, still face substantial shortages and gaps in the availability of doctors, nurses and midwives resulting in GPs having to care for an unusually high number of patients (World Health Organization, 2022). It is a vicious cycle – with higher numbers of registered patients, GPs are more prone to burn out and thus the shortage may become even worse. Moreover, GP practice owners were observed to score higher levels in EE, which is most likely related to administration workload. These findings are consistent with the French study (Dutheil et al., 2021).

According to the job demands-resources (JD-R) model, burnout arises when individuals experience incessant job demands and have inadequate resources available to address and reduce those demands. JD-R model considers PA a personal resource however, in case of its decrease, it becomes a workload and a burnout dimension (Maslach and Leiter, 2016). Diminished PA was the most prevalent dimension detected in our study. The dominance of this subscale may be related to the overall low opinion about GPs compared to other physicians in Czech society.

Given the response rate of 33.1 %, this study may be limited by selection bias, which may reflect in an underrepresentation of GPs with severe burnout. The response rates of e-mail surveys tend to approximate 25 % to 30 % (Fincham, 2008, Wu et al., 2022) and our previous experience suggests even lower rates (Štěpánek et al., 2023). On the other hand, the representation of females and males in the studied population is proportional to their representation in the entire Czech population of GPs, which testifies against selection bias. In addition, we have reached a determined sample size. The random selection of surveyed GPs across the nation constitutes the strengths of our study and, together with the above, enables generalizing the results to the entire Czech population of GPs.

## 5 Conclusions

Severe burnout was detected in 21.8 % of Czech GPs, with the most prevalent dimension of burnout being a lack of PA followed by EE and DP. The risk factors of severe burnout were male sex and an increased number of registered patients per GP. Burnout in GPs needs to be addressed to prevent progressive degeneration in primary care although there is little scientific evidence on how to improve GPs' well-being (Naehrig et al., 2021). Promoting personal resources as well as the perception of the importance of GPs in society would help in managing burnout in GPs. Emphasis needs to be placed on supporting additional GP recruitment to reduce the high patient burden on existing GP practices.

## CRediT authorship contribution statement

**Ladislav Štěpánek:** Conceptualization, Methodology, Writing – original draft. **Mihir Sanjay Patel:** Formal analysis, Writing – original draft. **Dagmar Horáková:** Writing – review & editing, Supervision. **Lubica Juríčková:** Investigation, Data curation, Writing – review & editing. **Svatopluk Býma:** Writing – review & editing, Project administration.

## Declaration of Competing Interest

The authors declare that they have no known competing financial interests or personal relationships that could have appeared to influence the work reported in this paper.

## Data Availability

Data will be made available on request.

## References

[b0005] Biber J., Ranes B., Lawrence S., Malpani V., Trinh T.T., Cyders A., English S., Staub C.L., McCausland K.L., Kosinski M., Baranwal N., Berg D., Pop R. (2022). Mental health impact on healthcare workers due to the COVID-19 pandemic: a U.S. cross-sectional survey study. J. Patient Rep. Outcomes.

[b0010] Brown P.A., Slater M., Lofters A. (2019). Personality and burnout among primary care physicians: an international study. Psychol. Res. Behav. Manag..

[b0015] Dreher A., Theune M., Kersting C., Geiser F., Weltermann B. (2019). Prevalence of burnout among German general practitioners: Comparison of physicians working in solo and group practices. PloS One.

[b0020] Dutheil F., Parreira L.M., Eismann J., Lesage F.X., Balayssac D., Lambert C., Clinchamps M., Pezet D., Pereira B., Le Roy B. (2021). Burnout in French General Practitioners: A Nationwide Prospective Study. Int. J. Environ. Res. Public Health.

[b0025] Fincham J.E. (2008). Response rates and responsiveness for surveys, standards, and the Journal. Am. J. Pharm. Educ..

[b0030] Karuna, C., Palmer, V., Scott, A., Gunn, J., 2022. Prevalence of burnout among GPs: a systematic review and meta-analysis. Br. J. Gen. Pract. 72, e316–e324. doi: 10.3399/BJGP.2021.0441. 10.3399/BJGP.2021.0441 PMC8869191 34990391

[b0035] Maslach C., Leiter M.P. (2016). Understanding the burnout experience: recent research and its implications for psychiatry. World Psychiatry.

[b0040] Maslach C., Jackson S.E., Leiter M.P. (1997).

[b0045] McCammon L.C., Gillen P., Kernohan W.G. (2023). Explaining and addressing the limitations in usefulness of available estimated prevalence figures relating to burnout in family doctors: Evidence from a systematic scoping literature review. J. Psychiatr. Res..

[b0050] Meier S.T., Kim S. (2022). Meta-regression analyses of relationships between burnout and depression with sampling and measurement methodological moderators. J. Occup. Health Psychol..

[b0055] Mojtahedzadeh N., Wirth T., Nienhaus A., Harth V., Mache S. (2021). Job Demands, Resources and Strains of Outpatient Caregivers during the COVID-19 Pandemic in Germany: A Qualitative Study. Int. J. Environ. Res. Public Health.

[b0060] Naehrig D., Schokman A., Hughes J.K., Epstein R., Hickie I.B., Glozier N. (2021). Effect of interventions for the well-being, satisfaction and flourishing of general practitioners-a systematic review. BMJ Open.

[b0065] Shen X., Xu H., Feng J., Ye J., Lu Z., Gan Y. (2022). The global prevalence of burnout among general practitioners: a systematic review and meta-analysis. Fam. Pract..

[b0070] Štěpánek L., Nakládalová M., Janošíková M., Ulbrichtová R., Švihrová V., Hudečková H., Sovová E., Sova M., Vévoda J. (2023). Prevalence of Burnout in Healthcare Workers of Tertiary-Care Hospitals during the COVID-19 Pandemic: A Cross-Sectional Survey from Two Central European Countries. Int. J. Environ. Res. Public Health.

[b0075] Verhoef N.C., Blomme R.J. (2022). Burnout among general practitioners, a systematic quantitative review of the literature on determinants of burnout and their ecological value. Front. Psychol..

[b0080] West C.P., Dyrbye L.N., Shanafelt T.D. (2018). Physician burnout: contributors, consequences and solutions. J. Intern. Med..

[b0085] World Health Organization. (2022, September 14). Ticking timebomb: Without immediate action, health and care workforce gaps in the European Region could spell disaster. Retrieved from https://www.who.int/europe/news/item/14-09-2022-ticking-timebomb--without-immediate-action--health-and-care-workforce-gaps-in-the-european-region-could-spell-disaster.

[b0090] Wu M.J., Zhao K., Fils-Aime F. (2022). Response rates of online surveys in published research: A meta-analysis. Comput. Hum. Behav. Rep..

