# Supplementary material for: High prevalence of burnout syndrome in Czech general practitioners: A cross-sectional survey
Source: Prev Med Rep. 2023 Nov 10;36:102502. doi: 10.1016/j.pmedr.2023.102502 (PMC10728438; doi:10.1016/j.pmedr.2023.102502)
Supplement: Supplementary data 1 [file mmc1.pdf]

# Corresponding Author's Declaration Form
Preventive Medicine

(This form is fillable with adobe software or equivalent.)

Manuscript title:	High prevalence of burnout syndrome in Czech general practitioners: a cross-sectional survey
Corresponding author:	Ladislav Štepanek
Additional authors in the order provided in the manuscript:	Mihir Sanjay Patel, Dagmar Horáková, Lubica Juríčková, Svatopluk Býma

The corresponding author must provide statements of authorship, originality, ethical compliance, conflicts of interest, and research funding on behalf of all authors of the manuscript.

Authorship and Originality:
The corresponding author warrants that all aforementioned authors fulfill the criteria of authorship as defined by the International Committee of Medical Journal Editors (ICMJE). The corresponding author further warrants that the work described in this manuscript has not been published before and is not (nor will be) under consideration elsewhere while under review in *Preventive Medicine*; that all authors approved the present submitted version and their institutions have no objections to the manuscript's contents.

Ethical compliance:
The corresponding author warrants that if the manuscript describes research on human subjects the necessary ethical approval (or exemption) has been obtained and is on file with the authors' institutions. For empirical research papers, add a statement of ethical compliance or exemption to the Methods section.

Conflict of interest:
The corresponding author declares the following financial or other relationships with companies or organizations that are stakeholders on the topic of the manuscript. For details read this fact sheet. Insert the same information in the manuscript as a final disclosure section.

☑	The authors have no conflicts of interest to disclose or
☐	The following authors report specific relationships that could be interpreted as implying a conflict (name author, nature of the relationship, and company or organization):

Funding source:
The corresponding author should declare all sources of funding and any involvement of study sponsors in the study design; collection, analysis and interpretation of data; the writing of the manuscript; or the decision to submit the manuscript for publication. If the study sponsors had no such involvement, this should be stated. Insert the same information in the manuscript as a final disclosure section.

☐	There were no sources of funding for this research
☑	The following authors report financial support (name author, granting organization, grant number, or company sponsoring the study):

Ladislav Stepanek - the Palacký University Olomouc Fund (RVO 61989592)

The corresponding author signed this statement on behalf of all coauthors to indicate that the above information is true, correct and complete.

Signature (both graphic and electronic validation signatures via software are acceptable): MUDr. Ladislav Štěpánek, Ph.D. Digitálně podepsal MUDr. Ladislav Štěpánek, Ph.D. Datum: 2023.05.03 19:54:14 +02'00'	Print name: Ladislav Štěpánek
	Date: May 3, 2023
